# Functional Tests of the Abdominal Wall Muscles in Normal Subjects and in Patients with Diastasis and Oblique Inguinal Hernias in a Pilot Study

**DOI:** 10.3390/jfmk9030164

**Published:** 2024-09-15

**Authors:** Dmitry Skvortsov, Andrei Cherepanin, Yulia Fadeeva, Andrey Timonin, Nataly Nosenko

**Affiliations:** 1 Research and Clinical Center of Specialized Types of Health Care and Medical Technology, 107031 Moscow, Russiajvdav@mail.ru (Y.F.); nataly1679@gmail.com (N.N.); 2 Institution Research Center of Nutrition and Biotechnology, 109240 Moscow, Russia

**Keywords:** abdominal hernia, functional test, electromyography, ultrasound investigation

## Abstract

Objectives: To identify typical patterns of abdominal wall muscle activation in patients with diastasis recti and inguinal hernias compared to controls during the Valsalva maneuver, voluntary coughing, and physical activity. Methods: The study included 15 subjects: 5 with diastasis recti, 4 with inguinal hernias, and 6 healthy controls. The functions of rectus abdominis (RA) and external oblique (OE) muscles were measured by surface electromyography (sEMG). Using ultrasound, the thicknesses of the RA, OE, internal oblique (IO), and transversus abdominis (TA) muscles were assessed as well as the echo intensity (EI) of RA and OE. Results: We found a significant effect of the type of abdominal wall pathology on the maximum sEMG amplitude (*p* = 0.005). There was a reliable trend in maximum sEMG amplitude, with the highest one in diastasis recti and a significantly lower one in inguinal hernias. Duncan’s test showed a significant difference in muscle thickness, both on the right and left sides, between patients with diastasis and controls, but only on the left side between patients with diastasis and those with inguinal hernia (*p* < 0.05). Conclusions: The abdominal wall pathology results in a change in the function and structure of the abdominal muscles, which can be detected using electromyography and ultrasound examination. The presence of diastasis recti is accompanied by an increase in bioelectrical activity and a decrease in thickness.

## 1. Introduction

The anterolateral abdominal wall is a strong and elastic multilayer structure that not only forms the limit of the abdominal cavity but also plays a key role in the function of external respiration, regulation of intra-abdominal pressure, and movements of the trunk and pelvis [1]. At the same time, the prevalence of conditions accompanied by abdominal wall insufficiency—hernias, diastasis recti, muscular dystrophy, and sarcopenia—is extremely high. Thus, the cumulative incidence of inguinal hernias, estimated in a population of more than 51 million people, is 7.7%, while among people over 60 years of age, it is up to 30% [2,3]. The true incidence of recti diastasis is unknown. However, according to some data, it reaches 39% in older parous women and 52% in urogynecological patients in menopause [4]. In 2013, Filip Muysoms proposed the term Post-Partum Abdominal Wall Insufficiency (PPAWI), which includes diastasis recti and develops in more than 30% of women after pregnancy [5].

Assessment of the functional state of the abdominal muscles using EMG has been carried out since the 1950s of the 20th century with most studies showing a decrease in the amplitude of muscle bioelectric activity (BEA) in patients with abdominal hernias [6] with a predominance of changes on the affected side [7]. However, the assessment of the BEA of the abdominal muscles in combination with the identification of characteristic activation patterns during various tests is of no less importance [8]. Apparently, an imbalance in the activity of the abdominal muscles and the diaphragm plays a significant role in hernia formation, which leads to sharp fluctuations in intra-abdominal pressure during breathing and, as a result, progressive overstretching with thinning of the abdominal wall in its weak points [9]. In 2021, Shah et al. suggested that there is a certain ratio of BEA of the rectus and oblique abdominal muscles during forced expiration, which turns out to be impaired following laparotomy [10].

Since the work of Nobel laureate Archibald Hill, it has been known that the size and relative position of muscle fibers are of decisive importance in muscle functioning [11]. The presence and prevalence of histological changes in the abdominal muscles in patients with abdominal hernias remains a matter of debate. According to Jensen et al., there are no significant histological changes in the rectus abdominis muscles in postoperative hernia patients with a medial size of fascial defect of more than 10 cm. The density of capillaries, the distribution of muscle fibers, and the degree of fibrosis and adipose infiltration correspond to those in individuals with an intact abdominal wall, based on which the authors concluded that the functional insufficiency of the rectus abdominis muscles in patients with hernias is determined only by anatomical displacement and loss of the medial insertion [12]. In the meantime, according to de Silva et al., hernioplasty leads to hypertrophy of the rectus abdominis and oblique muscles, which indicates their previous atrophic changes [13].

Structural muscle changes can be detected by ultrasound, which shows sensitivity reaching 92% when using fully quantitative image analysis methods [14]. Echo intensity (EI) corresponds to the degree of fibrosis and adipose degeneration and is more closely related to muscle function rather than its size. A correlation between muscle EI and the strength of isometric contraction, ability to accelerate, and developed power has been proven. However, most published papers report studies of the muscles of the back and limbs [15].

Considering the availability of ultrasound, it is extremely important to search for typical structural features of the anterior abdominal wall muscles, reflecting their biomechanical properties and pathological potential. The purpose of this study is to identify typical patterns of the anterior abdominal wall muscle activation in patients with recti diastasis and inguinal hernias compared to controls during voluntary coughing and physical activity, as well as to compare the obtained data with structural changes in muscles during an ultrasound examination.

Functional provocative tests provide valuable clinical information. For the abdominal muscles, potential reproducible voluntary tests include coughing and tensing the abdominal muscles while raising the head and shoulder from a supine position. Can these tests detect differences in muscle function between healthy people and patients with abdominal hernias?

## 2. Materials and Methods

### 2.1. Subjects

The study included 15 subjects: 5 with recti diastasis, 4 with inguinal hernias, and 6 controls (Table 1). Participants were examined between June and November 2022. The width of diastasis was estimated based on the distance between the rectus abdominis muscles at the midline 3 cm above the umbilical ring, ranging from 5 to 11 cm (Grade 2–3). In all patients with inguinal hernias, the pathology was bilateral. All subjects signed an informed consent before being included in the study. The study protocol was approved by the local ethical committee of the Federal Research and Clinical Center of specialized types of health care and medical technology of the Federal Medical-Biological Agency (Extract 3 from protocol No. 1_2022 14/01/2022).

The exclusion criteria included injuries of the spine, pelvis, or head; a history of neuromuscular and rheumatic diseases; and chronic use of glucocorticosteroids. All subjects were right-handed.

### 2.2. Motor Tests Used

The motor function of the abdominal wall muscles was assessed using the Valsalva maneuver, voluntary cough, and raising the shoulder girdle in the supine position. The Valsalva maneuver was monitored by the cessation of blood flow in the superficial femoral vein in the upper third of the thigh in color Doppler mode [16].

**Figure 1:**
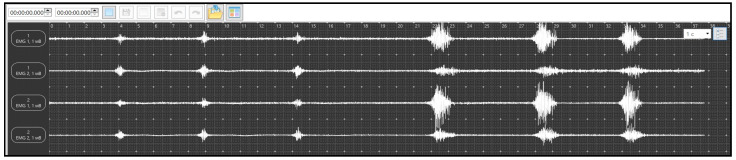
Samples of EMG activity: the three consecutive excitations on the left are the Cough Test and the three consecutive excitations on the right are the Head Lift Test. The upper channel is the right rectus abdominis, the second from the top is the right obliquus externus, the third from the top is the left rectus abdominis, and the lower is the left obliquus externus.

Before the record initiation, the subjects underwent training. The training was monitored visually using EMG. The subject lay on the couch with his head raised and his hands behind his head. In this position, he produced a single cough. The nature of the action was determined by EMG and hearing. The training consisted of the subject making one movement while exhaling. The criterion for completing the training was the moment when the subject made single cough exhalations, which were identical in sound and amplitude, while the EMG pattern was symmetrical in terms of increasing and decreasing activity (Figure 1). Similar training was carried out for the head lift exercise. The subject abruptly lifted his head (with his hands remaining behind his head) from the couch and returned it to the starting position. The criterion for completing the training was considered the moment when the lifts and the corresponding EMG activity of the muscles were of the same amplitude and equal in time and the EMG activity was symmetrical in increasing and decreasing (Figure 1).

During recording, the subject made three coughing movements and three head lifts at intervals of 2–3 s.

### 2.3. Study of EMG Activity

Sensors NEUROSENS (Neurosoft, Ivanovo, Russia) were used to study the EMG activity of the abdominal wall muscles. The sensors contain an inertial navigation module and two EMG channels with a data recording frequency of 2000 Hz and data transmission via a Wi-Fi channel. Channel synchronization error not less than <5 ms. Registration was carried out using disposable surface electrodes SWAROMED (Kyoto, Japan).

**Figure 2:**
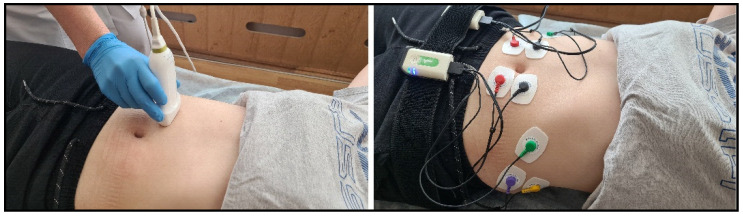
Ultrasound examination of muscles (**left**) and EMG examination (**right**).

The rectus and external oblique abdominal muscles were studied symmetrically on the right and left sides. The following designations were used: RA for the rectus abdominis muscle and OE for the obliquus externus abdominis muscle (Figure 2).

**Figure 3:**
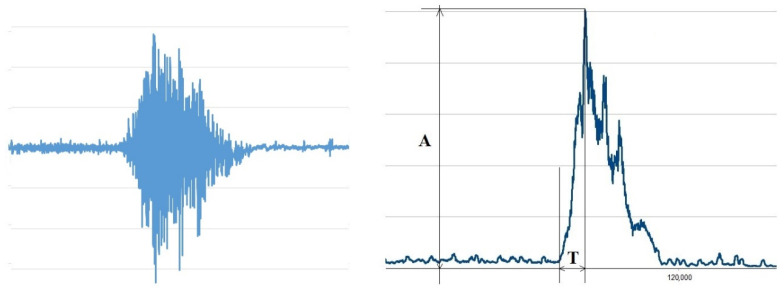
Native EMG (**left**) and measured parameters (**right**). Where are “A”—amplitude (μV), “T”—time (ms).

The received data were converted into an Excel spreadsheet for further processing. The native EMG curve was full-wave rectified and smoothed with a constant of 200 ms (Figure 3).

For each action, its amplitude was determined in μV (A) and the time to reach the maximum amplitude in s (T) using the rectified and smoothed EMG. The onset of contraction was determined using the Lasserson et al. approach, according to which the onset of contraction was considered to be an increase in EMG amplitude of more than two standard deviations from activity at rest [17].

### 2.4. Ultrasound Imaging of the Abdominal Muscles

Ultrasound imaging was carried out in B-mode on a scanner Philips Lumify (Koninklijke, Philips Medical Systems, Bothell, WA, USA) using a linear probe with a frequency range of 4–12 MHz. The subjects were positioned supine with their legs straightened. The maximum thickness of the following muscles was measured as follows: RA, OE, OI, and TA. The thickness of the rectus muscle was measured 2 cm above the navel; the sensor was displaced laterally from the midline until the maximum muscle size was captured in the center of the image. The thickness of the muscles of the lateral wall of the abdomen was measured along the anterior axillary line in the middle of the distance between the costal margin and the anterior superior iliac spine.

Quantitative analysis of grayscale images of the rectus and external oblique abdominal muscles was performed using ImageJ SW (ImageJ 2.0.0-rc-43/1.52n/Java 1.8.0_411), a freely available program (National Institute of Mental Health, Bethesda, MD, USA). Ultrasound scanner settings were constant throughout the studies. Taking into account the dependence of the EI of the underlying formations on the overlying ones, quantitative analysis was carried out only for superficially located muscles, i.e., RA and OE.

The main research question is whether the objective methods used to study muscle function in the functional test are sensitive to detect changes in patients with hernias of the anterior abdominal wall. Can normal variants be distinguished from pathological ones? Another question is whether this functional test can provide objective differences between two different clinical types of hernia.

### 2.5. Statistical Processing

Data processing was carried out using StatSoft, Inc. (2014). STATISTICA (data analysis software system), version 12 (www.statsoft.com). Data were presented as mean ± standard deviation (SD). Given the small sample sizes, groups were compared using several non-parametric methods of analysis. In particular, the method of rank coding of the higher activity, rather than the rectus muscles results of direct measurements of the parameters under study, was used to conduct a rank one and multivariate analysis of variance, the Spearman correlation R. Differences were considered significant at *p* < 0.05. To assess the accuracy of the research, the research accuracy indicator, i.e., the level of accuracy, φ, was calculated. The level of research accuracy for the corresponding indicator was determined taking into account the specified type I and II error probabilities, α = 0.05 and β = 0.1, according to the calculation formula zβ − zα = ∆ ∙ √n/sx; φ = Δ/μ, where zβ and zα are standardized indicators of the general population with type I and II error probability, respectively; Δ, which is the difference between the arithmetic means of the sample for the case of the null hypothesis and its alternative; μ, which is the arithmetic mean under a true null hypothesis; and φ, which is an accuracy level. The accuracy level for a reliably interpreted data analysis protocol shall be below 0.2. When establishing the factor response, multiple comparison analysis was carried out using Duncan’s test. A comparison of correlations was based on a comparison of the obtained correlation values by the strength of the connection under the following classification: from 0.7 to 1—strong, from 0.69 to 0.4—medium, and from 0.39 to 0—weak connection.

## 3. Results

### 3.1. EMG Study

During the Valsalva test, a low-amplitude EMG curve (10–30 μV) was recorded, which did not allow for reliably distinguishing a contraction from the noise and determining its onset.

The maximum EMG curve amplitude during coughing and the contraction time parameters are presented in Table 2.

In patients with diastasis, RA and OE activity was higher than in those with inguinal hernia. In patients with an inguinal hernia, activity was also higher than in the controls. However, the time and duration were different. Thus, so far, the only evident finding is increased muscle activity in patients with diastasis. Another detail to be mentioned is that the oblique muscles showed much higher activity than the rectus muscles.

The oblique muscles did not show such superior activity during the Lift Test (Table 3). However, both also showed higher activity compared to the controls. However, there were exceptions, i.e., left OE in patients with an inguinal hernia was significantly lower compared to not only those with diastasis but controls as well.

The time and duration during this test were higher because the movement itself was longer. These parameters are characterized by asymmetry. The duration of contraction of the rectus and external oblique muscles during cough was similar among study groups (right rectus, *p* = 0.7; left rectus, *p* = 0.5; right external, *p* = 0.2; and left external, *p* = 0.4).

### 3.2. Ultrasonography

Absolute values of abdominal muscle thickness and EI are represented in Table 4 and Table 5.

### 3.3. Analysis of Variance

#### 3.3.1. EMG

When using the rank coding of the dependent variable EMG–response, by calculating the mathematical expectation on the entire EMG spectrum, a rank two-factor analysis of variance was carried out for each subject. The following values were obtained: *p* = 0.8 for the factor of the abdominal wall pathology type (factor A), *p* = 0.91 for the factor muscle type (factor B), and *p* = 0.99 for the inter-factor correlation. The reason for this effect may be due to an insignificant factor load on the dependent variable or significant variations in the study sample. To determine the possible impact on sample stabilization, the coefficient of variation (C%) was calculated. For this model, C% was 748%, which practically makes the expectation of a reliable response for such a model inconsistent. To increase the accuracy of the study, another initial parameter for calculating the dependent variable was chosen, i.e., the determination of the maximum weighted EMG signal (MWM). To determine this indicator, the spectrum of each subject was ranked in ascending order. Then, the minimum number of MWM signals was calculated, the calculation of the arithmetic mean of which would have a coefficient of variation lower than 0.35. This number was 100 signals in a ranked series in descending order of MWM. The arithmetic mean of MWM for each patient was subjected to rank coding followed by analysis according to the 3 × 4 plan. A significant effect of the type of abdominal wall pathology on maximum EMG activity was revealed (*p* = 0.005), with an insignificant difference between RA and OE (*p* = 0.544) and no inter-factor correlation (*p* = 0.99).

To determine an adequate level of significance within the framework of this study, provided that the type II error was below 0.15 and the accuracy of the study was 0.2, a calculation was carried out, taking into account the number of subjects. As a result, a significance level of 0.1 was obtained. With this value, the type II error and the accuracy of the study made up 0.14 and 0.18, respectively, which allows for considering this analysis as experimentally reliable (the results of the statistical response showed a good correlation with the target response of the study). To establish the influence of factor A, a non-parametric multiple comparison test—Duncan’s test—was used. With its use of factor A, the following results were obtained: controls showed a significant difference from patients with diastasis (*p* < 0.1), while the latter showed a significant difference from those with an inguinal hernia. It shall be noted that there is a reliable trend identified through a distribution-free study in relation to the muscle maximum EMG activity in various types of pathology: the highest activity was in diastasis and the significantly minimal one was in inguinal hernias.

#### 3.3.2. US Examination

Muscle thickness was analyzed using a rank two-way analysis of variance within the framework of the 3 × 4 plan. Factors A and B for muscle thickness on the right made up *p* = 0.047 and *p* < 0.0001, respectively; AB for inter-factor correlation made up *p* = 0.82. Factors A and B for muscle thickness on the left made up *p* = 0.039 and *p* < 0.0001, respectively; AB for inter-factor correlation made up *p* = 0.84. The significance level was 0.05. In this case, the type II error was 0.125 and the accuracy level was 0.2. Thus, both factors A and B were significant. The inter-factor correlation AB was not significant, showing that the rated factors were independent.

Duncan’s test showed a significant difference between muscle thickness both on the right and left in controls and patients with diastasis, while in patients with diastasis and those with inguinal hernia, it was different on the left (*p* < 0.05).

Echo intensity on the right showed no significant response for the rated factors and the inter-factor correlation AB. The significance level was 0.1. In this case, the type II error was 0.142 and the accuracy level was 0.2. Factors A and B for echo intensity on the left made up *p* = 0.011 and *p* = 0.02, respectively; AB for the inter-factor correlation made up *p* = 0.41. The significance level was 0.1. In this case, the type II error was 0.142 and the accuracy level was 0.2. Both factors A and B were significant. The inter-factor correlation AB was not significant, showing that the rated factors were independent.

Duncan’s test showed a significant difference for factor A between patients with diastasis and controls (*p* < 0.05).

#### 3.3.3. Method Comparability

Three-way rank analysis of variance was performed to examine the correlation type between muscle thickness and MWM. The factors rated were the following: A for pathology type, B for muscle type, and C for analysis type. The number of levels of factors A, B, and C was 3 (diastasis, control, and inguinal), 4 (1–4 muscles), and 2 (ultrasound and EMG), respectively. The experimental design was 2 × 3 × 4. During the examination of muscle thickness (cm) for the dependent variable US thickness, EMG and factors A, B, and C made up *p* = 0.22, *p* < 0.0001 and *p* = 0.65, respectively. AB, BC, AS, and ABC for inter-factor correlations made up *p* = 0.98, *p* = 0.00016, *p* = 0.0002, and *p* = 0.75, respectively. The significance level was 0.05. In this case, the type II error was 0.125 and the accuracy level was 0.15. Thus, factor A was insignificant and factor B was significant. The inter-factor correlation AB was insignificant, BC and AC were significant, and ABC was insignificant, showing that there was a significant relationship between the factors’ pathology type and analysis type, as well as muscle type and analysis type. Thus, this study allows for stating that a full-fledged analysis of the correlation between muscle type and pathology type must necessarily be carried out in a complex interpretation of the methods used to obtain a response. In this case, the data analysis and interpretation allow for a much larger amount of information.

Duncan’s test showed the following for factor B: RA/OE, RA/TA, OE/OI, and OI/TA were significantly different (*p* < 0.05). It should be taken into account that, according to factor strength, muscles had a significant biological significance for this dependent variable.

#### 3.3.4. Correlation Analysis

A Spearman’s correlation for four types of muscles showed a significantly strong (linearized) correlation between RA/OI and OE/TA (r was 0.89 and 0.8, respectively). An average correlation was noted for RA/OE (r = 0.58); however, this level of correlation could be mediated by the influence of external factors or be formal.

To establish the mutual influence of muscles, an analysis was carried out using partial multiple correlations. A high and direct correlation was revealed between RA/OI and OE/TA (r = 0.87), which was not significantly affected by other muscle types. It was also found that the RA/OE correlation was significantly affected by other muscle types (significantly by OI with r = 0.82).

The results of the correlation analysis of the maximum amplitude of the straightened and smoothed EMG curve for the Cough and Lift Tests, the duration of EMG activity during the Cough Test, and sonographic characteristics of the rectus and external oblique muscles on the right and left are shown in Table 6.

In controls, a strong correlation was revealed between all of the studied parameters; for patients with diastasis, not only cough strength but also cough duration for OE (r = −0.53) was significantly different; a weak connection was also registered for OE thickness and RA echo intensity. In all other cases, the connection was strongly similar to that in controls. There were also significant differences in correlations for inguinal hernia, i.e., weak and inversely proportional cough duration for OE. In all other cases, the correlation was strong. Thus, it was established that the factor pathology type has a significant biological significance for the functioning of the muscles. Various pathologies result in muscle functioning pattern changes. Diastasis is characterized by more significant differences.

When comparing different (electrophysiological and ultrasound) characteristics of the same muscle, a different nature of the correlation was noted for controls, patients with diastasis, and patients with an inguinal hernia. A similar result is possible either with extreme variability of such associations or with stable characteristics of each muscle. A small sample size does not allow for establishing this fact within the framework of the current study.

## 4. Discussion

The primary goal of this study was to evaluate the BEA of the abdominal muscles in comparison with their sonographic features. There were three groups of subjects: patients with recti diastasis, patients with inguinal hernias, and controls. A higher EMG activity of the studied muscles in the group of patients with diastasis was revealed compared to controls, with a simultaneous decrease in muscle thickness and a less significant increase in echo intensity. A violation of the correlation between the duration of contraction of the external oblique muscles during cough was demonstrated, which was more pronounced in patients with diastasis. Moreover, the ratios of the thickness of the anterior abdominal wall muscles were revealed, which were not significantly affected by the pathology type considered.

The strength of muscle contraction depends on the muscle size, its activation pattern, its constituent fiber nature, and the proportion of non-contracting cellular elements. Muscle size can be characterized by measuring its thickness, cross-sectional area, and volume; the proportion of connective tissue elements by assessing echo intensity; and muscle activation nature by recording the biopotentials of motor units that trigger the physiological processes of muscle contraction. The question of the intercorrelation of these indicators, their correlation with the force of contraction, and the nature of their changes under various physiological and pathological conditions has been actively studied in recent years. Thus, during power loads, the root-mean-square values of the EMG-curve amplitude increase first, then the muscle thickness increases, and finally, the echo intensity changes. For the muscles of the extremities, the correlation between the mean square EMG values and the strength of the maximum voluntary muscle contraction was revealed [18] but no such correlation was revealed for the anterior abdominal wall muscles. One of the reasons for that seems to be the significant interaction of agonists and antagonists, which leads to an increase in the stabilizing role of the muscle, rather than to the generation of torque [19].

The study of the function of the anterior abdominal wall muscles includes, among other things, an assessment of their role in the process of external respiration. Exhalation has long been considered as a passive process. For the first time, BEA of the abdominal muscles during exhalation was recorded by Campbell et Green using skin electrodes in 1953 [20]. In 1987, using needle electrodes, Goldman et al. showed that during quiet breathing, the abdominal muscles are inactive, BEA can be recorded only during cough, the Valsalva maneuver, and at the end of expiration with an increase in the minute volume of breathing [21]. The rectus and the lateral abdominal wall muscles have different effects on the thorax shape: the rectus muscles reduce its anteroposterior size, making it more elliptical, while isolated stimulation of the external oblique muscle reduces the transverse dimension, making it more cylindrical [22]. During forced exhalation, the BEA of the oblique muscles is higher than that of the rectus muscles [23] and the activity of OI is higher than OE [9]; meanwhile, the maximum expiratory pressure correlates more with RA thickness and the peak expiratory flow with OE thickness [24]. When coughing, a predominance of activity of the oblique muscles over the rectus muscles has also been described [25], which corresponds to the results obtained in the current study. The anterior abdominal wall muscles optimize the diaphragm function by tensioning the lower edge of the costal arch. There is a direct relationship between the magnitude of intra-abdominal pressure and the anterior abdominal wall muscle activity [22,26]. The increase in BEA of the abdominal wall muscles revealed in the current study, which was most pronounced in patients with diastasis, probably reflects the degree of increase in their intra-abdominal pressure. Similar changes—an increase in the amplitude of the EMG curve in combination with a decrease in muscle thickness during US—have been described in women during pregnancy [27].

The obtained values for the duration of voluntary cough are more than twice as large as published data [20], probably because the test was carried out while lying down and not sitting. However, LoMauroetAliverti showed that despite mechanical changes in the supine position (cranial displacement of the diaphragm, decrease in lung volume, stretching of the muscles involved in exhalation, and decrease in the elasticity of the abdominal wall), the maximum expiratory flow rate and muscle activation during coughing do not depend on body position [28]. The revealed disruption of correlations between the duration of the external oblique muscle activation during coughing in patients with the anterior abdominal wall pathology, being more pronounced in diastasis, probably reflects their functional separation when performing coordinated motor acts.

The values of the thickness of the anterior abdominal wall muscles in controls obtained during the study corresponded to the published data [29]. A linear relationship between the thickness of RA and OI, as well as OE and TA, were revealed. At the same time, the pathology type did not significantly affect muscle thickness. Such conservatism of US parameters is emphasized by the constant order of their thickness decreasing (RA > OI > OE > TA), shown on large samples of healthy subjects [30].

Gabrielsen et al. described the benefits of texture analysis of ultrasound images of abdominal muscles as a method for assessing the degree of adipose infiltration [29]. According to computed tomography, atrophy and adipose infiltration of the abdominal and pelvic muscles increase the risk of hernia formation [31,32]. However, it is unclear whether these changes are part of the pathogenesis of hernia disease or whether they reflect the general physical condition of the patient [33]. In this study, an increase in the echo intensity of the rectus and external oblique muscles in patients with rectus diastasis was revealed, probably due to an increase in the content of adipose and fibrous components in them. However, existing studies do not allow stating with certainty which histological changes in muscle tissue are reflected in an increase in its echo intensity during US examination [15].

The main limitation of the study is the small sample size, which did not allow for taking into account the dependence of the measured values on gender and anthropometric parameters, the size of diastasis, and the side of the hernia defect in inguinal hernias.

In addition, the study has inherent disadvantages that are typical for the surface recording of an electromyogram, i.e., the dependence of the signal amplitude on the thickness of the subcutaneous adipose tissue layer, as well as distortion of the BEA signal from neighboring muscles and ECG artifacts.

Given the role of the abdominal wall in maintaining posture, the factors affecting the abdominal muscle thickness are diverse and difficult to analyze. Thus, the dependence of the abdominal muscle thickness and the magnitude of diastasis of the rectus muscles on the presence of the Achilles tendon pathology [34] and pain in the lower back [35] has been demonstrated.

For a more complete informative development of this topic, the studies of the nature of the functional distribution of the EMG spectrum of muscles with different types of hernias of different types of muscles are planned, which involves the use of multidimensional research methods.

The proposed functional test can be informative for studying muscle dysfunction in patients with abdominal hernias and assessing their subsequent condition after surgical treatment at various times.

## 5. Conclusions

The abdominal wall pathology results in a change in the function and structure of the abdominal muscles, which can be detected using electromyography and ultrasound examinations. The presence of diastasis recti is accompanied by an increase in bioelectrical activity, a decrease in thickness, and, to a lesser extent, an increase in echo intensity of the anterior abdominal wall muscles, as well as a disconnection in the time parameters of contraction of the external oblique muscles during coughing. There are certain correlations of the anterior abdominal wall muscle thickness, which are little affected by pathology. The proposed functional test can be used to study muscle function in patients with abdominal hernias.

## Figures and Tables

**Table 1:** Anthropometrics of the study groups. Variables presented as median [interquartile range].

Parameter	Control	Diastasis	Ing. Hern	*p*
Age (years)	29 [26; 35]	39 [38; 43]	73 [64; 75]	0.02
Gender (M:F)	4:2	2:3	4:0	
Height, (m)	1.6 [1.7; 1.9]	1.6 [1.6; 1.7]	1.8 [1.8; 1.8]	0.07
Weight, (kg)	82 [65; 106]	64 [51; 80]	82 [77; 88]	0.3
BMI (kg/m^2^)	27.1 [22.8; 31.3]	26.0 [20.2; 27.0]	25.5 [23.5; 28.1]	0.5
Subcutaneous adipose tissue thickness, (cm)	2.1 [1.1; 3.1]	1.0 [1.0; 1.4]	2.0 [1.8; 2.0]	0.8

**Table 2:** sEMG parameters during the Cough Test. Variables presented as median [interquartile range]. The sEMG amplitude (A), (μV), and time to reach the maximum amplitude (T), s, of rectus abdominis (RA) and external oblique (EO) muscle activities did not differ between the studied groups.

Muscle	Parameter	Control	Diastasis	Ing. Hern	*p*
RA right	A	27.5 [25.6; 27.9]	85.0 [75.0; 97.7]	44.0 [37.5; 48.5]	0.13
	T	0.3 [0.2; 0.7]	0.3 [0.3; 0.8]	0.3 [0.3; 0.4]	0.70
RA left	A	40.4 [32.3; 54.2]	83.7 [75.0; 97.7]	39.4 [31.3; 46.3]	0.75
	T	0.3 [0.2; 0.4]	0.3 [0.2; 0.5]	0.2 [0.2; 0.3]	0.53
OE right	A	63.7 [43.7; 94.8]	102.3 [83.0; 154.7]	59.0 [44.5; 92.0]	0.14
	T	0.4 [0.3; 0.5]	0.7 [0.7; 0.7]	0.4 [0.5; 0.5]	0.23
OE left	A	68.3 [45.8; 117.6]	94.3 [93.7; 129.0]	72.2 [49.4; 106.3]	0.40
	T	0.3 [0.2; 0.4]	0.4 [0.3; 0.7]	0.4 [0.4; 0.5]	0.39

**Table 3:** sEMG amplitudes of rectus abdominis (RA) and obliquus externus (OE) muscles during the Lift Test, (μV). Variables presented as median [interquartile range].

Muscle	Side	Control	Diastasis	Ing. Hern	*p*
RA	Right	193 [153; 214]	269 [124; 282]	167 [118; 218]	0.68
	Left	161 [121; 196]	184 [160; 207]	156 [107; 205]	0.89
OE	Right	110 [43; 192]	83 [83; 168]	88 [71; 124]	0.99
	Left	119 [32; 204]	124 [70; 173]	83 [66; 105]	0.89

**Table 4:** Absolute values of muscle thickness and total thickness of lateral abdominal wall (SUM), (cm), of rectus abdominis (RA), obliquus externus (OE), obliquus internus (OI), and transversus abdominis (TA). Variables presented as median [interquartile range].

Muscle	Side	Control	Diastasis	Ing. Hern
RA	Right	1.3 [1.1; 1.5]	0.7 [0.7; 0.8]	0.9 [0.8; 1.1]
	Left	1.2 [1.1; 1.4]	0.8 [0.7; 1.0]	0.8 [0.7; 1.0]
OE	Right	0.5 [0.4; 1.0]	0.5 [0.5; 0.6]	0.5 [0.5; 0.6]
	Left	0.6 [0.5; 0.9]	0.5 [0.5; 0.5]	0.5 [0.4; 0.6]
OI	Right	0.9 [0.6; 1.1]	0.6 [0.6; 0.8]	1.2 [1.0; 1.3]
	Left	1.0 [0.7; 1.1]	0.6 [0.6; 0.7]	1.1 [1.0; 1.2]
TA	Right	0.5 [0.3; 0.6]	0.3 [0.3; 0.5]	0.4 [0.3; 0.6]
	Left	0.5 [0.3; 0.6]	0.3 [0.3; 0.4]	0.5 [0.4; 0.6]
SUM	Right	1.9 [1.5; 2.5]	1.5 [1.4; 1.8]	2.1 [2.0; 2.2]
	Left	2.0 [1.7; 2.6]	1.4 [1.3; 1.6]	2.1 [1.7; 2.5]

**Table 5:** Abdominal Muscle Echo intensity (arbitrary units). RA—rectus abdominis muscle. OE—obliquus externus muscle. Ing. Hern—inguinal hernia. Variables are presented as median and upper and lower quartile. Variables are presented as median [interquartile range].

Muscle	Side	Control	Diastasis	Ing. Hern
RA	Right	30 [21; 45]	58 [53; 64]	41 [36; 52]
	Left	34 [26; 38]	51 [49; 53]	40 [29; 54]
OE	Right	60 [49; 67]	62 [50; 73]	66 [64; 71]
	Left	48 [42; 53]	51 [47; 77]	65 [61; 70]

**Table 6:** Correlation analysis of the studied indicators on the right and left. RA—rectus abdominis muscle. OE—obliquus externus muscle.

Parameter Item	Cough		Cough Duration		Body Lift		Muscle Thickness		Echo Intensity	
	RA	OE	RA	OE	RA	OE	RA	OE	RA	OE
Control	0.74	0.78	0.96	0.95	0.69	0.95	0.93	0.86	0.95	0.86
Diastasis	0.74	0.84	0.76	−0.53	0.91	0.86	0.83	0.03	0.33	0.93
Ing. Hern	0.99	0.99	0.88	−0.22	0.97	0.95	0.92	1.00	0.95	0.98

## Data Availability

Any additional information or data can be requested from the authors.
